# Restoring cell‐cell communication networks to enhance cognition and reduce inflammation in Alzheimer’s disease

**DOI:** 10.1002/alz70859_106608

**Published:** 2025-12-26

**Authors:** Nareh Tahmasian, Tina Beckett, Ke Cao, Mary Hill, Matthew Mandrozos, Chao Wang, JoAnne McLaurin

**Affiliations:** ^1^ University of Toronto, Toronto, ON Canada; ^2^ Sunnybrook Research Institute, Toronto, ON Canada; ^3^ Biological Sciences, Sunnybrook Research Institute, Toronto, ON Canada

## Abstract

**Background:**

Alzheimer’s disease (AD) is a devastating neurodegenerative disease estimated to affect over 55 million people worldwide. It is characterized by a progressive loss of neurons leading to deterioration of memory and cognitive ability. An innovative strategy has recently emerged to replace lost neurons by converting another type of brain cell, astrocytes, into neurons. This involves injecting a virus carrying specific ‘reprogramming’ transcription factor genes, driven by the astrocyte GFAP promoter. Preliminary work in our laboratory suggests that this approach successfully improves learning and memory in a rat model of AD by not only increasing the number of new neurons but also reducing neuroinflammation. We hypothesize that these newly formed neurons not only replace lost ones but also communicate with nearby cells to promote protection and repair.

**Methods:**

First, we utilized single‐cell RNA‐sequencing(scRNA‐seq) technology to characterize how AD affects cell‐cell communication changes in the hippocampus in human AD patients and a rat model of AD. Next, we injected virus carrying reprogramming transcription factors directly into the hippocampus of AD rats and used scRNA‐seq and spatial transcriptomics (Visium platform) to study how these cell‐cell communication networks are affected by astrocyte‐to‐neuron conversion.

**Results:**

Compared to controls, the hippocampus in human AD and the AD rat model had significant changes in signaling, such as an increase in neuregulin signaling between neurons, which is known to modulate synaptic plasticity. We found that astrocyte‐to‐neuron conversion significantly altered cell‐cell communication networks in the AD rat hippocampus, including reversing many AD‐associated signaling patterns.

**Conclusions:**

Astrocyte‐to‐neuron conversion reverses several AD‐associated signaling pathways, which may be part of the underlying mechanism for its beneficial effects on cognition and neuroinflammation. This work not only elucidates some of the cascading molecular effects of astrocyte‐to‐neuron conversion but also highlights pathways that can be directly enhanced or inhibited to potentiate protective or regenerative effects.

